# Hypotheses on the Potential of Rice Bran Intake to Prevent Gastrointestinal Cancer through the Modulation of Oxidative Stress

**DOI:** 10.3390/ijms18071352

**Published:** 2017-06-24

**Authors:** Bernard M. H. Law, Mary M. Y. Waye, Winnie K. W. So, Sek Ying Chair

**Affiliations:** The Nethersole School of Nursing, The Chinese University of Hong Kong, Shatin, The New Territories, Hong Kong; bernardlaw@cuhk.edu.hk (B.M.H.L.); mary-waye@cuhk.edu.hk (M.M.Y.W.); winnieso@cuhk.edu.hk (W.K.W.S.)

**Keywords:** rice bran, oxidative stress, gastrointestinal cancer, cancer prevention, antioxidants

## Abstract

Previous studies have suggested the potential involvement of oxidative stress in gastrointestinal cancers. In light of this, research efforts have been focused on the potential of dietary antioxidant intake to prevent gastrointestinal cancer through the modulation of oxidative stress. Rice bran, a by-product of rice milling, has been shown to contain an abundance of phytochemicals, which are dietary antioxidants. To date, a number of studies have shown the antioxidative effect of rice bran intake, and some demonstrated that such an effect may contribute to gastrointestinal cancer prevention, largely through the antioxidative properties of rice bran phytochemicals. In addition, these phytochemicals were shown to provide protection against cancer through mechanisms linked to oxidative stress, including β-catenin-mediated cell proliferation and inflammation. The present article provides an overview of current evidence for the antioxidative properties of rice bran and its phytochemicals, and for the potential of such properties in cancer prevention through the oxidative-stress-linked mechanisms mentioned above. The article also highlights the need for an evaluation of the effectiveness of rice bran dietary interventions among cancer survivors in ameliorating oxidative stress and reducing the level of gastrointestinal cancer biomarkers, thereby establishing the potential of such interventions among these individuals in the prevention of cancer recurrence.

## 1. Introduction

Gastrointestinal cancers, defined as those occurring at various sites that include the colon, rectum, stomach, liver, pancreas and oesophagus, form one of the most common cancer groups worldwide. In 2012, more than 3.8 million new cases of gastrointestinal cancer were reported worldwide, and its incidence is expected to increase to almost 6.2 million by 2030 [1]. Therefore, there is a pressing need for the development of effective strategies to prevent gastrointestinal cancers.

Previous research has focused on seeking a better understanding of the molecular mechanisms of gastrointestinal carcinogenesis. During the years of research, a number of physiological factors have been identified concerning such mechanisms. For example, it was suggested that colorectal cancer (CRC) can be caused by intestinal inflammation and microbial dysbiosis [2], while stomach cancer was found to be the result of *Helicobacter Pylori* infections [3,4]. However, among all the various cancer-associated physiological factors, oxidative stress appears to be one of the most studied to date. In the light of this, research has been directed towards the potential use of dietary antioxidants, the edible compounds known to reduce oxidative stress, in cancer chemoprevention. Likewise, previous research has also focused on whether the intake of certain foods generally consumed by humans conferred a protective effect against oxidative stress. Rice bran, a by-product of rice milling previously shown to contain a variety of bioactive compounds that exhibit antioxidant properties, is one of the food sources that have been widely studied for their antioxidant and anticancer potential. The aim of the present paper is to provide an overview of current data on the anti-oxidative effect of rice bran, and the potential mechanisms of how such effect may lead to gastrointestinal cancer prevention. The review will first provide a brief account of how oxidative stress occurs, and the evidence supporting the hypothesis that ameliorating oxidative stress can reduce cancer risks. Studies that show the antioxidative effect of rice bran intake, together with those suggesting that rice bran intake may prevent cancer through the modulation of oxidative stress, will then be reviewed. Finally, the potential mechanisms involved in this chemo-preventive effect will be discussed in the context of findings concerning the protective function against oxidative stress of the bioactive compounds present in rice bran.

## 2. Oxidative Stress

**Figure 1:**
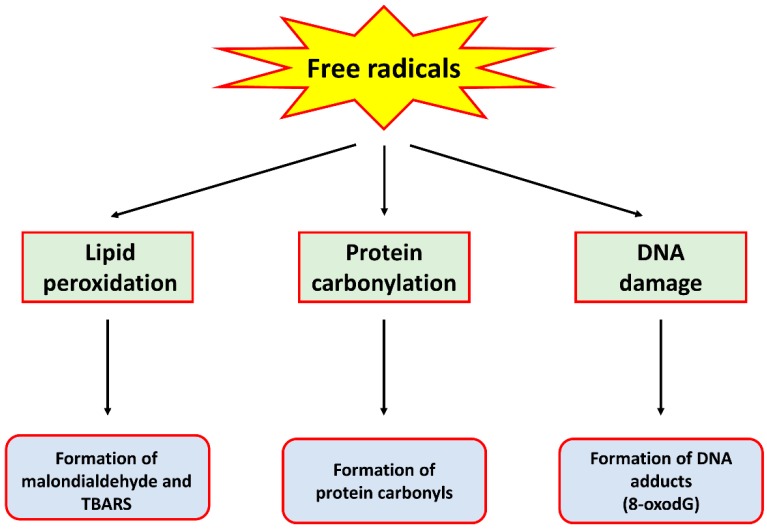
A schematic diagram summarising the detrimental effects of free radicals on biomolecules.

Oxidative stress is a condition where the rate of production of free radicals far exceeds that of their removal by antioxidant enzymes, therefore causing an accumulation of the former. These free radicals, generally termed reactive oxygen species (ROS) and reactive nitrogen species (RNS), are produced through various metabolic processes, including cellular respiration [5] and immune reactions by immune cells [6]. While the presence of low levels of these free radicals is beneficial to cellular functions such as the regulation of signalling pathways [7], they in fact have deleterious effects on cells when they are produced at high levels. Indeed, ROS and RNS have been shown to cause oxidation and/or nitration of lipids, proteins and DNA, resulting in damage to these biomolecules. For example, hydroxyl radical, the product of a reaction between ROS such as superoxide anion and hydrogen peroxide (H_2_O_2_), can cause lipid peroxidation, protein carbonylation and the formation of DNA adducts such as 8-oxo-7,8-dihydro-2’-deoxyguanosine (8-oxodG), all of which are markers of oxidative stress [8]. An RNS such as peroxynitrite, formed by the reaction between superoxide anion and nitric oxide, has also been shown to cause damage to these biomolecules [9]. A summary of the detrimental effects of these free radicals is provided in Figure 1.

To counter the damaging effects of these free radicals, cells utilise a repertoire of antioxidant enzymes and molecules to remove such excess free radicals and maintain a healthy redox balance. For example, superoxide dismutase (SOD) can scavenge superoxide anion and convert it to hydrogen peroxide, which, by the action of catalase or glutathione peroxidase (GPx), would be further detoxified. These enzymes would therefore be able to prevent the formation of the damaging ROS and RNS mentioned above. Furthermore, the antioxidant molecule glutathione, with the help of glutathione-S-transferase (GST), would help lower the level of oxidative stress by detoxifying the products of lipid peroxidation and DNA oxidation [10]. Taken together, ROS and RNS can be produced through certain physiological processes, with their levels kept in check by the cellular antioxidant system. Oxidative stress occurs if these free radicals are produced in excess, or if the antioxidants cannot keep up with their removal, eventually leading to an accumulation of oxidative damage to the biomolecules in the cells.

## 3. The Contribution of Oxidative Stress in Cancer

To date, numerous studies have provided evidence for the role of oxidative stress in cancer progression, and the link between the two has been extensively reviewed [11,12,13,14,15,16]. Moreover, studies of cancer-promoting intestinal bacteria have also suggested the involvement of oxidative stress in gastrointestinal cancers. For example, *Helicobacter pylori* infection, long known to cause gastric cancer [3,4], can produce ROS such as superoxide and peroxynitrite [17]. *Enterococcus faecalis*, a bacterial species whose faecal abundance was found to be increased in colorectal cancer patients [18], was also shown to produce superoxide [19] and hydroxyl radicals [20]. These studies suggest the involvement of bacteria associated with gastrointestinal cancer in exacerbating oxidative stress, and provide further evidence for the role of oxidative stress in gastrointestinal cancer.

In fact, previous studies have provided multiple lines of evidence that oxidative stress can increase cancer risks. For instance, the ROS produced by the colonic bacteria *Enterococcus faecalis* were shown to induce potentially carcinogenic mutations of colonic DNA, with their mutagenic effect being prevented by the action of catalase [21]. Products of lipid peroxidation such as malondialdehyde can react with DNA and form “exocyclic DNA adducts”, which has been suggested to contribute to gastrointestinal carcinogenesis [22]. Moreover, inflammatory bowel disease, a condition characterised by an increased level of oxidative stress [23], was found to be associated with increased colorectal cancer risks [24]. Consistent with this finding, higher urinary 8-oxodG levels, indicative of a high level of oxidative stress-associated DNA damage, were observed in CRC patients [25,26]. In contrast, it has been suggested in some in vitro and in vivo studies that dietary antioxidants have a potential for gastrointestinal cancer prevention. For example, luteolin, a bioflavonoid with antioxidant properties, was shown to exhibit anti-proliferative and pro-apoptotic effects in a colon cancer cell line [27]. Cocoa’s antioxidant properties were demonstrated in a mouse model of colitis-associated cancer, and it was also suggested as a potential cancer chemo-preventive agent [28]. Studies providing evidence that dietary antioxidants may inhibit cancer progression were recently reviewed by Galadari et al. [15].

However, controversies do exist as to the anticancer effects of the intake of antioxidants, or the lowering of oxidative stress levels. Indeed, clinical trials of dietary antioxidant intake among humans have failed to yield consistent results on whether the intake of dietary antioxidants confers protective effects against cancer. Previously, a meta-analysis had shown that antioxidant supplementation had no effect on CRC incidence and recurrence, although the supplementation of a vitamin C and vitamin E combination had a slight inhibitory effect on CRC [29]. While methodological differences could be a factor contributing to the observed discrepancies between studies, the poor bioavailability of the ingested antioxidants was also suggested as a likely factor for such discrepancies [15]. As suggested by Galadari et al., the ingested antioxidants are often poorly absorbed and quickly metabolised [15], thus the antioxidants used in certain studies may not have enough opportunity to exert their beneficial effects on the human body before they are metabolised and excreted. This phenomenon could well have led to the large variation in their effects on cancer prevention and the inconsistency of data derived from these clinical trials.

Despite the discrepancies in the findings of different studies, the well-established involvement of oxidative stress in cancer, including gastrointestinal cancers, has indeed sparked immense interest among scientists in further research into the use of certain dietary supplements known to contain abundant compounds with antioxidant properties for gastrointestinal cancer prevention. Rice bran is one of such dietary supplements that has been gaining increasing research interest over the past decade in its potential for use in cancer chemo-prevention.

## 4. Rice Bran

Rice bran is produced as a by-product of the milling process of rice, a staple food largely cultivated in Asia and South America and widely consumed by humans worldwide. After harvesting, rice needs to undergo a series of milling processes to remove the outer layers of the grains in order to produce the white rice that is generally consumed as part of the human diet. One such process involves the removal and processing of the bran, the coating that covers whole grain rice produced after the removal of rice hull, thereby creating rice bran. However, the world’s population does not generally use such rice milling by-products as part of their daily diet. Rice bran contains lipoxygenases that break down the fatty acids present in the bran, which after an extended period of storage would eventually result in a detrimental effect on its flavour [30], making it less edible. Despite such drawbacks, rice bran actually contains an abundance of bioactive compounds, generally known as phytochemicals, that lead to better health and the chemoprevention of cancer. Previous studies have in fact suggested the role of rice bran in the chemoprevention of gastrointestinal cancer, and also proposed potential mechanisms for how its intake can confer a measure of chemo-prevention. These include the inhibition of cell proliferation, anti-inflammation and modification of the intestinal microbiota. These mechanisms have been demonstrated in previous studies and reviewed elsewhere [31,32].

## 5. Evidence for the Anti-Oxidative Effects of Rice Bran

**Figure 2:**
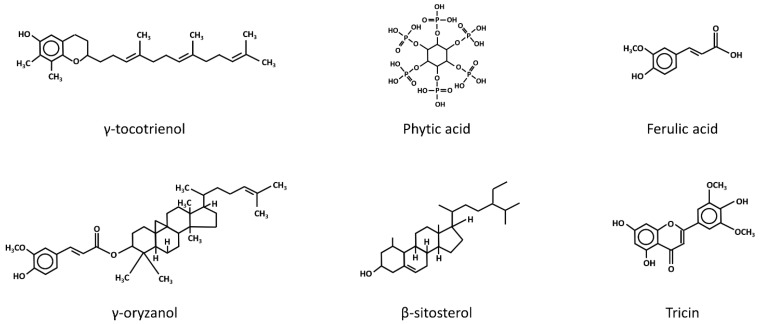
The chemical structures of the anti-oxidative phytochemicals present in rice bran. β-Sitosterol and tricin are members of the phytosterol and flavonoids families.

In addition to the potential mechanisms mentioned above, the modulation of oxidative stress levels has also been suggested as an alternative cancer chemo-preventive mechanism of rice bran intake. In fact, a repertoire of phytochemicals present in rice bran, including tocotrienols, phytic acid, γ-oryzanol, ferulic acid, phytosterols and flavonoids (Figure 2), has been shown to possess antioxidant properties [33]. Moreover, nutrients such as vitamin B, which is abundantly present in rice bran, appear to exhibit a preventive effect against oxidative stress. A recent case-control study demonstrated that a reduced intake of vitamin B was associated with an increased level of oxidative stress, as evidenced by the higher level of lipid peroxidation and glutathione (GSH) depletion in subjects exhibiting low vitamin B intake [34]. This was also supported by the findings of a previous animal study, where mice deficient in vitamin B6 displayed characteristics of increased oxidative stress, including an increased lipid peroxidation level [35]. In other words, the phytochemicals and nutrients present in rice bran may play a significant role in conferring the protective effect of rice bran intake against oxidative stress.

Consistent with this hypothesis, a number of in vivo and in vitro studies have also demonstrated the benefits of rice bran intake in the amelioration of oxidative stress. Boateng et al. had previously demonstrated that rice bran dietary supplementation for rats with colon tumours would result in an almost two-fold increase in the activity of GST, which detoxifies the oxidative products of biomolecules, in the colon [36]. More recently, supplementing the diet of rats with aqueous enzymatic extract from rice bran was also found to enhance the activity of antioxidant enzymes such as SOD, catalase and glutathione peroxidase, with the level of oxidative damage of lipids and proteins reduced compared with the control rats [37]. Moreover, such treatment was also found to cause a significant reduction in the production of superoxide radicals and a decreased expression of reduced nicotinamide adenine dinucleotide phosphate (NADPH) oxidase, an enzyme capable of superoxide production, in rats [38].

In addition, increased free radical scavenging and antioxidant activity were also demonstrated in several in vitro models upon treatment with rice bran extracts. For example, treatment of HL-60 cells, a human leukaemia cell line, with ethanolic extracts of brown rice and rice bran resulted in a reduction in superoxide production, coupled with a decrease in the extent of lipid peroxidation in these cells [39]. A dose-dependent increase in the ROS and nitric oxide scavenging activity was also observed in a glioma cell line treated with methanolic extract of Njavara rice bran [40]. Recently, Lee et al. showed that pre-treatment of a liver cancer cell line (HepG2) with rice bran extract obtained from black rice reversed the detrimental effects of chemically-induced oxidative stress. Further observations upon the treatment of the cells with the extract include the suppression of ROS generation, an increase in reduced glutathione content and the inhibition of lipid peroxidation [41], indicating an improvement in the antioxidant status of these cells after treatment.

Furthermore, rice bran extracts were demonstrated to reduce oxidative stress in some non-cancer cell lines upon treatment. For example, fermented rice bran extract was shown to inhibit ROS generation in cultured adipocytes subjected to H_2_O_2_-induced oxidative stress [42]. Further, pre-treatment with rice bran extract on rats’ heart cells exposed to H_2_O_2_ was also found to increase both the activity and the level of expression of antioxidant enzyme catalase [43]. Such studies confirm the above in vitro studies regarding the inhibitory effect of rice bran intake on ROS production in cancer cell lines.

In addition, rice bran oil, extracted from rice bran, has been demonstrated to exhibit antioxidant potential in several in vivo studies. Administering rice bran oil to rats subjected to chemically-induced oxidative stress was found to reduce their level of lipid peroxidation. This was coupled with a slight increase in the activity of catalase, SOD and GPx, although this effect was not prominent [44]. Likewise, Riceberry bran oil, extracted from the bran of a new variety of Thai rice, was shown to exhibit similar effects on lipid peroxidation and antioxidant enzyme activity upon supplementation for diabetic rats on a high-fat diet [45]. In a study using rats treated with azoxymethane, a carcinogen known to induce colon cancer in animal models, administration of rice bran oil was shown to result in 50% higher GST activity in the rats’ colons, when compared with the control rats [46]. Further, in streptozotocin-induced diabetic rats, supplementation of rice bran oil in their diet led to amelioration of mitochondrial DNA damage in the liver, kidney and pancreas of these rats, as evidenced by the reduction in the level of 8-oxodG present in these organs [47]. Taken together, the above studies suggest that rice bran intake elicits a protective effect against oxidative stress, and this effect is likely to be attributed to multiple mechanisms, including the modulation of antioxidant enzyme activity and expression, inhibition of oxidative damage to biomolecules and direct scavenging of ROS. A summary of the evidence demonstrating the anti-oxidative effects of rice bran intake is presented in Table 1.

## 6. The Anti-Oxidative Properties of Rice Bran Contribute to Cancer Chemo-Prevention

As discussed above, numerous studies have provided evidence for a link between oxidative stress and cancer initiation and progression, and potential mechanisms have been proposed to explain how ROS can be carcinogenic. With multiple studies pointing towards the potential of rice bran intake in the relief of oxidative stress, it is therefore tempting to speculate that the antioxidant properties of rice bran may have an inhibitory effect on tumour growth and cancer progression. Several studies have demonstrated that the antioxidative effects of rice bran intake may contribute to cancer prevention. An early study [48] revealed that supplementation with tocotrienol-rich fraction isolated from rice bran oil would reduce the level of lipid peroxidation and protein oxidation caused by exposure of rats to diethylnitrosamine (DEN)/2-acetylaminofluorene (AAF), a combination of chemicals used to induce stomach cancer. The effect was accompanied by a reduction in the activity of alkaline phosphatase, an enzymatic marker for gastrointestinal cancers [50], in the liver of these rats. Moreover, in mice bearing Ehrlich tumour cells, administering modified arabinoxylan rice bran was found to inhibit the growth of Ehrlich tumours. This inhibitory effect was accompanied by an improvement in the antioxidant status of these mice, including reduced lipid peroxidation, increased activity and expression of antioxidant enzymes and elevated glutathione levels [49]. These findings can further be supported by the observation that brewers’ rice, a rice product comprising broken rice, rice bran and rice germ, may also exhibit anticancer effects through its antioxidant properties. Tan et al. [51] showed that supplementation with brewers’ rice would reverse the oxidative effects of azoxymethane treatment in rats. These effects include a decrease in SOD expression and an elevation in the levels of nitric oxide and malondialdehyde (MDA), a product of lipid peroxidation. This observation therefore suggests that brewers’ rice supplementation would be likely to confer protection against azoxymethane-induced colon cancer in these rats through inhibition of oxidative stress. Interestingly however, one study [52] suggested that the anticancer effect of the intake of rice bran was not attributable to the amelioration of oxidative stress, but rather to the inhibition of inflammation. The authors treated fibrosarcoma QR-32 cells with fermented brown rice and rice bran with *Aspergillus oryzae*, a supplement that had previously been shown to inhibit azoxymethane-induced colon carcinogenesis [53]. They found that such treatment reduced the infiltration of inflammatory cells into the tumours and inhibited the expression of pro-inflammatory genes, but failed to reduce the level of 8-oxodG formation. Although the discrepancy in findings between this study and others is unclear, it is possible that the antioxidative effects of the phytochemicals in the rice bran used in the study may have prevented the further oxidation of 8-oxodG, rather than the formation of 8-oxodG itself. It was suggested that 8-oxodG is not the only product of DNA oxidation as a result of oxidative stress, as 8-oxodG is able to be further oxidised into spiroiminodihydantoin deoxyribonucleoside under such conditions [54]. A previous study also showed that incubation of DNA with more than 100 µM tea polyphenols, which were known to exhibit antioxidant properties, exhibited no effect on 8-oxodG levels in the presence of hypochlorous acid, a product formed under oxidative stress conditions [55]. The authors argued that the tea polyphenols, present in high concentrations, had directed their antioxidant action at preventing the conversion of 8-oxodG to spiroiminodihydantoin deoxyribonucleoside as a result of oxidative stress, rather than the formation of 8-oxodG. Therefore, the observations made by Onuma et al. [52] could be the result of inhibiting the further oxidation of 8-oxodG by the rice bran phytochemicals, and that is why they were not able to observe a decrease of 8-oxodG as a result of rice bran treatment. In any case, while it is true that rice bran intake may prevent carcinogenesis through anti-inflammatory mechanisms, it is not appropriate to rule out the role of oxidative stress modulation in rice bran’s anticancer effect.

In fact, the chemo-preventive properties of rice bran, as indicated in the above studies, could be largely attributed to the antioxidant effect of the aforementioned phytochemicals present in rice bran, including phytic acid and phenolic acids. To date, several in vivo studies have provided evidence for the role of these phytochemicals in the inhibition of gastrointestinal cancers through the modulation of oxidative stress. For example, phytic acid was shown to increase GST activity and reduce the level of lipid peroxidation in a rat model of hepato-carcinogenesis, and these effects were coupled with a decreased appearance of hepato-carcinogenesis markers [56]. This suggests that phytic acid may suppress liver tumourigenesis through amelioration of oxidative stress. In another study, the ability of phytic acid to increase GST activity, which resulted in a decrease in the number of colon tumours in a rat model of CRC, was also demonstrated [57]. In addition, an early study [58] showed that the administration of ferulic acid, a phenolic acid abundantly present in rice bran, to azoxymethane-treated rats could decrease the number of aberrant crypt foci (ACF), an early sign and a potential marker for the development of colon cancer [59]. This phenomenon may be related to the increase in GST activity in these treated rats, thereby demonstrating the role of antioxidant properties of ferulic acid in colon cancer prevention. Further, supplementation of p-methoxycinnamic acid, a rice bran phenolic acid, was found to reverse the decrease in the levels of both antioxidant enzymes and molecular antioxidants caused by the treatment of dimethylhydrazine, a chemical for the induction of colon cancer, in rats. These effects were also accompanied by a decrease in the formation of ACF in these rats [60]. Interestingly, an in vitro study by the same research group showed that p-methoxycinnamic acid treatment on a human colon adenocarcinoma cell line (HCT-116) would actually induce oxidative stress in these cells, demonstrated by an increase in ROS production and a concomitant decrease in the activity of antioxidant enzymes. The authors also postulated that these pro-oxidative effects of p-methoxycinnamic acid treatment could confer protection to these cells against cancer through apoptotic induction [61]*.* The reasons for the discrepancy in the effects of p-methoxycinnamic acid treatment shown by these two studies remain as yet unclear. However, the difference in the models used (in vivo vs. in vitro) and the dosage of the p-methoxycinnamic acid used in both studies could constitute the contributory factors for this discrepancy. Nevertheless, these studies show that the antioxidative effects of rice bran are likely to be exerted by the phytochemicals known to exhibit antioxidant properties that are abundantly present in rice bran. These studies also provide evidence that the protective effect of these phytochemicals against gastrointestinal cancer is at least partly attributable to their antioxidant properties. Table 2 provides a summary of the studies showing the anti-oxidative effects of rice bran phytochemicals and their associated effect on gastrointestinal cancer prevention.

Overall, the studies presented above suggested an involvement of the regulation of oxidative stress levels in the chemo-preventive effect of rice bran intake, likely due to the antioxidant properties of the phytochemicals present in rice bran. Further studies showing an association between the reduction of oxidative stress markers (such as lipid peroxidation, DNA oxidation and protein carbonyl formation) and inhibition of tumour growth in cellular and animal models of further types of gastrointestinal cancers, such as stomach and pancreatic cancers, would be useful in supporting this hypothesis.

## 7. Other Potential Oxidative-Stress-Related Mechanisms for the Chemo-Preventive Effect of Rice Bran Intake

With previous research demonstrating an implication of oxidative stress in carcinogenesis, research efforts have been directed to the elucidation of the mechanisms in which oxidative stress can lead to gastrointestinal cancer. Notably, oxidative stress was shown to be linked to a variety of molecular mechanisms or pathways where their aberrant activation would lead to cancer. These include inflammation and signalling pathways involving mitogen-activated protein kinase (MAPK), p53, β-catenin, Class O forkhead box protein (FOXO) and NF-κB [62,63]. It is therefore tempting to speculate that the intake of antioxidants that ameliorate oxidative stress and perhaps interfere with these pathways would have a protective effect against gastrointestinal cancers.

As indicated above, the ability of the phytochemicals present in rice bran to modulate the level of oxidative stress would at least partly contribute to the anticancer effect of rice bran intake. However, previous studies suggest that these phytochemicals may also confer such anticancer effects through other mechanisms, including the reduction of inflammation and the regulation of β-catenin signalling, a pathway associated with cell proliferation and tumour metastasis. As mentioned, both β-catenin signalling and inflammation are linked to oxidative stress, leaving us with the question of whether the phytochemicals, which are antioxidants themselves, may confer their anticancer effects through these mechanisms using their antioxidant properties. In other words, would the modulation of oxidative stress by these phytochemicals be able to control aberrant β-catenin signalling and inflammation, the two known contributors to cancer? This section of the review provides an overview of the studies that provide evidence suggesting the relationship between oxidative stress and β-catenin signalling and inflammation, and also the evidence for the effect of certain rice bran phytochemicals on the regulation of β-catenin signalling and inflammation.

### 7.1. β-Catenin Signalling

β-catenin is a protein that acts as a mediator of the effect of Wnt signalling, a signalling pathway known to contribute to cell proliferation and whose aberrant activation is implicated in cancer [64]. It forms part of a transcriptional complex that, when activated, would lead to the expression of genes involved in cell survival and proliferation (cyclins, c-myc and survivin) and tumour metastasis (metalloprotease), in the nucleus of cells. Indeed, multiple lines of evidence suggest that oxidative stress can lead to increased β-catenin activity. Increased activation of NADPH oxidase I (NOX1), a superoxide-producing enzyme known to be associated with colon cancer [65], has been shown to inhibit the degradation of β-catenin via a decrease in its phosphorylation by glycogen synthase kinase 3 (GSK3). On the contrary, knockout of NOX1 would result in a loss of β-catenin activity and a reduction in cyclin D1 expression [66]. These findings therefore demonstrate that increased superoxide production, causing oxidative stress, would promote β-catenin-mediated gene expression. In addition, in a renal carcinoma rat model, Liu et al. [67] showed that chronic oxidative stress can activate β-catenin signalling, leading to an increased expression of the target genes of β-catenin such as cyclin D1. Further, treatment of a human colorectal cancer cell line (SNU-407) with H_2_O_2_ was found to increase the activity of Akt, a potential upstream regulator of β-catenin [68], leading to an increased nuclear localisation of β-catenin and increased expression of cyclin D1 [69]. Consistent with these findings, antioxidant treatment on cells was shown to inhibit Wnt/β-catenin signalling through the suppression of β-catenin dephosphorylation, thereby promoting the degradation of β-catenin in the proteasome and inhibiting β-catenin-mediated gene expression [70]. Taken together, oxidative stress can promote cell proliferation through the increased activity of β-catenin and the resulting increased expression of pro-proliferative genes.

**Figure 3:**
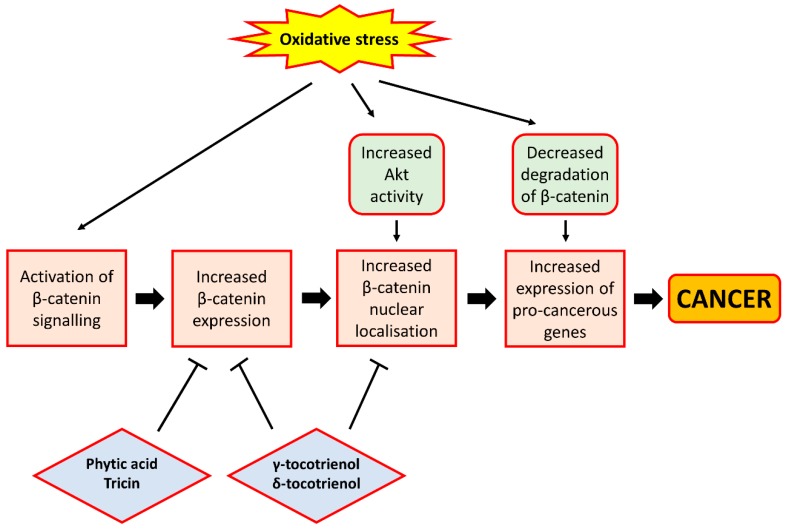
The relationship between oxidative stress and β-catenin signalling. Oxidative stress can lead to activation of β-catenin signalling. This can be achieved through the ability of H_2_O_2_ to activate Akt, leading to the increased nuclear localisation of β-catenin, or the ability of superoxide to inhibit the degradation of β-catenin, causing increased expression of pro-cancerous genes such as cyclin D1, c-myc and survivin. On the contrary, rice bran phytochemicals such as phytic acid, tricin and tocotrienols can inhibit β-catenin signalling, largely through the inhibition of β-catenin expression and nuclear localisation. These phytochemicals therefore exhibit cancer chemo-preventive properties by preventing β-catenin from activating the expression of pro-cancerous genes. In the figure, arrows indicate ‘promotion’ or ‘lead to’, and bar-headed lines indicate ‘inhibition’.

Several studies involving cancer cell lines have provided evidence that certain phytochemicals present in rice bran would help suppress cell proliferation through their interference in Wnt/β-catenin signalling, primarily through a reduction in β-catenin expression. γ-Tocotrienol and δ-tocotrienol, both of which are vitamin E derivatives, were found to reduce the level of expression and nuclear localisation of β-catenin in colon cancer cell lines, leading to reduced expression of cyclin D1, c-myc and survivin in these cells [71,72]. Moreover, Rajendran et al. [73] also demonstrated in hepatocellular carcinoma cell lines that γ-tocotrienol treatment could cause a down-regulation of the activity and expression of signal transducer and activator of transcription 3 (STAT3), a transcription factor shown to be activated by Wnt/β-catenin signalling [74], leading to the reduced expression of pro-proliferative genes. Consistent with these findings, another study showed that tocotrienol-rich fraction extracted from palm oil could reduce β-catenin expression and cyclin D1 and survivin expression in colon cancer xenografts inoculated in mice [75], thereby demonstrating the ability of tocotrienols to inhibit cell proliferation through the down-regulation of the Wnt/β-catenin pathway. In addition, tricin, a bioflavonoid abundantly present in rice bran, was also shown in colon cancer cells to reduce β-catenin levels, thereby reducing the expression of markers for cancer stem cells that may promote tumour growth [76]. Likewise, phytic acid administration among rat models of CRC was also found to decrease β-catenin expression, an effect shown in separate studies to be accompanied by a decreased expression of Ki67, a marker for cell proliferation [77] and decreased formation of aberrant crypt foci and incidence of colon tumours [78]. These studies indicate that rice bran phytochemicals may interfere with Wnt/β-catenin signalling via a reduction in β-catenin expression, which results in a decrease in the proliferative ability of cells owing to the inhibition of β-catenin-mediated expression of genes implicated in cell proliferation. Moreover, Ahmed et al. [79] found that γ-tocotrienol can reverse epithelial-to-mesenchymal transition of tumours, a process implicated in the initiation of tumour metastasis, through the inhibition of β-catenin signalling, although the study involved the use of breast cancer cells. This study suggests that certain rice bran phytochemicals may also prevent tumour metastasis and cancer progression through the suppression of β-catenin signalling, owing to the ability of β-catenin to induce the expression of pro-metastatic genes such as metalloproteases, although further studies using gastrointestinal cancer cell lines or animal models are required to confirm this. A depiction of the relationship between oxidative stress and β-catenin signalling, and the effect of rice bran phytochemicals on β-catenin signalling, is presented in Figure 3.

### 7.2. Inflammation

Inflammation has long been considered to contribute to tumour progression [80]. Previous reviews have also suggested the involvement of inflammation in a variety of gastrointestinal cancers [81,82,83,84], therefore indicating the potential of anti-inflammatory strategies in the prevention of gastrointestinal cancers. In fact, previous studies have generated ample evidence for the inter-relationship between oxidative stress and inflammation. Oxidative stress was shown to be implicated in inflammatory bowel diseases (IBD), which are characterised by inflammation in the gastrointestinal tract [85]. This observation is further demonstrated by studies showing that an increased level of 8-oxodG levels and protein carbonyls were found in patients with Crohn’s Disease and ulcerative colitis, both of which are major types of IBD [13,86]. Moreover, ROS had previously been demonstrated to be a contributor to the inflammatory process. For example, hydroxyl radical and superoxide may interact with lipids present in cell membranes and form 4-hydroxynonenal (4-HNE), a molecule that promotes inflammation by provoking the release of pro-inflammatory mediators from immune cells [87]. H_2_O_2_ may also contribute to inflammation through its ability to activate NF-κB [88], a transcription factor that has long been considered to play a role in intestinal inflammation through mediating the expression of inflammatory cytokines such as interleukin-6 and TNF-α [89,90]. Moreover, mice deficient in nuclear factor erythroid 2-related factor 2 (Nrf2), a transcription factor responsible for the expression of antioxidant genes [91], would be more susceptible to the development of intestinal inflammation under the effect of dextran sulphate sodium [92], a drug commonly used to induce colitis in animal models. This finding further supports the involvement of oxidative stress in intestinal inflammation.

While oxidative stress can lead to inflammation, an increase in the condition could also play a role in exacerbating oxidative stress. Immune cells such as neutrophils, a major player in inflammation, would express NADPH oxidase, which can produce superoxide [93]. Once activated, the increased superoxide production by these neutrophils would further worsen the local oxidative status, thereby forming a vicious cycle of oxidative stress and inflammation. The bi-directional exacerbating effect between oxidative stress and inflammation may perhaps explain the involvement and coexistence of both conditions in gastrointestinal diseases, such as CRC. Overall, oxidative stress can potentially contribute to inflammation, largely through the effect of ROS on promoting the activity of immune cells in producing and releasing pro-inflammatory mediators. Moreover, oxidative stress and inflammation are liable to exacerbate each other in inflammation-based diseases, providing further evidence for the inter-relationship between these two molecular events.

**Figure 4:**
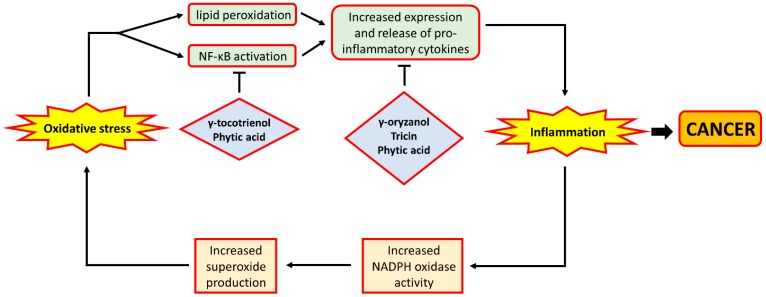
The relationship between oxidative stress and inflammation. ROS such as hydroxyl radical and superoxide anion may cause increased lipid peroxidation, leading to the formation of 4-HNE which may promote the release of pro-inflammatory cytokines. Likewise, H_2_O_2_ may activate NF-κB, a transcription factor that mediates the expression of pro-inflammatory cytokines. This leads to increased level of inflammation which was demonstrated to be implicated in carcinogenesis. Moreover, during inflammation, neutrophils would step up the production of superoxide anion by NADPH oxidase. The increased superoxide production would further exacerbate oxidative stress, thereby forming a vicious cycle. On the other hand, rice bran phytochemicals were shown to amoeliorate inflammation, either through the inhibition of NF-κB activity or the reduction of pro-inflammatory gene expression. In the figure, arrows indicate ‘promotion’ or ‘lead to’, and bar-headed lines indicate ‘inhibition’.

The anti-oxidative phytochemicals present in rice bran have also been shown in a number of studies to possess anti-inflammatory properties. Dietary supplementation of γ-oryzanol, an antioxidant in rice bran with potent radical scavenging activity, was found to reduce inflammation in rats by suppressing the production of pro-inflammatory cytokines released by activated macrophages [94]. In a mouse model of colon cancer, tricin supplementation was demonstrated to lower the expression of the pro-inflammatory tumour necrosis factor-α in the colonic mucosa of the mice [95]. Further, in studies involving the use of human colon cell lines, tricin treatment was shown to inhibit the activity of cyclo-oxygenase, an enzyme responsible for the generation of pro-inflammatory mediators such as prostaglandins [96,97], as well as the inhibition of the production of interleukin-6 [98]. Phytic acid treatment of colon cancer cell lines was also shown in separate studies to reduce the expression of IL-6 [99] and TNF-α [100], together with their receptors, in these cells. Such observations were also confirmed in vivo among rats fed with a high-fat diet [101]. In addition to the above effects, phytic acid treatment of cells was also found to increase the expression of I-κB [102], an inhibitor of NF-κB, which was indicated above to be a mediator of both inflammation and cell proliferation. More recently, a study also showed that phytic acid treatment of Caco-2 cells was able to reverse the pro-inflammatory effects of the treatment with inflammatory mediators through the down-regulation of the expression of inducible nitric oxide synthase (iNOS) [103], an enzyme whose over-expression was observed in patients with IBD [104]. Furthermore, in vitro γ-tocotrienol treatment was shown to inhibit the activity of NF-κB, leading to a decrease in the expression of pro-inflammatory and pro-proliferative genes, including cyclo-oxygenase 2, cyclin D1 and c-myc [105,106]. The above observations indicate that phytochemicals present in rice bran appear to play an important role in the reduction of inflammation, primarily through the down-regulation of pro-inflammatory gene expression. A schematic depiction of how oxidative stress interacts with inflammation, and how rice bran phytochemicals may inhibit inflammation, is shown in Figure 4.

Taken together, the phytochemicals present in rice bran can exhibit a chemo-preventive effect not only through the regulation of oxidative stress, but also by interfering with other pro-tumourigenic cellular mechanisms that were shown to be related to oxidative stress, such as the β-catenin-mediated expression of pro-proliferative genes and inflammation. Further studies are likely to be valuable in elucidating whether these phytochemicals exhibit their interference in these cellular mechanisms through their inherent antioxidative properties. Such studies might provide further evidence for the role of the reduction of oxidative stress in cancer chemoprevention.

## 8. Future Research Directions

To date, the majority of studies investigating the antioxidative and anticancer effects of rice bran and its phytochemicals have mainly involved the use of cancer cell lines and animal models of cancer. However, studies that look at the effect of rice bran intake among humans on the relief of oxidative stress are currently lacking. As indicated earlier, oxidative stress is implicated in the pathogenesis of certain gastrointestinal cancers, and increased oxidative stress has been observed in gastrointestinal cancer patients. It is therefore tempting to speculate that the amelioration of oxidative stress could potentially be effective in the inhibition of gastrointestinal tumour growth, and therefore the prevention of cancer recurrence among gastrointestinal cancer survivors. With rice bran containing abundant phytochemicals that exhibit antioxidant properties, research on the use of rice bran dietary interventions among gastrointestinal cancer survivors would be of great value.

As suggested by Galadari et al. [15], dietary antioxidants are likely to be poorly absorbed and quickly metabolised, raising the question of what dosage of rice bran needs to be ingested for the phytochemicals it contains to achieve a maximum antioxidative effect. Therefore, in studies involving rice bran dietary interventions, the required dosage of rice bran for the optimal antioxidative effect would first need to be established among gastrointestinal cancer survivors, by monitoring the changes in the biomarkers for oxidative stress before and after the intervention. These biomarkers may include serum levels of thiobarbituric acid reactive substances and 8-oxodG, both of which are oxidation products of bio-molecules generated as a result of oxidative stress. Rice bran dietary interventions using the optimal dosage determined may then be performed to establish whether they confer an inhibitory effect on tumour progression, through the measurement of various tumour markers at different time points during the intervention, such as carcino-embryonic antigen [107]. Such studies would provide further evidence for the anticancer effect of rice bran through the reduction of oxidative stress, and establish the potential for the use of rice bran dietary interventions in cases of gastrointestinal cancers recurring among the cancer survivors.

## 9. Conclusions

Rice bran is a dietary supplement that is known to contain abundant phytochemicals with potent antioxidant properties. Evidence has been emerging from previous in vivo and in vitro studies that these phytochemicals can inhibit the growth of gastrointestinal tumours, potentially through the amelioration of oxidative stress, the inhibition of cell proliferation and the reduction of inflammation. Such data would provide useful evidence for making a case for investigating the potential of rice bran intake for gastrointestinal cancer prevention in humans. Nevertheless, few studies to date have looked at the effects of rice bran intake in ameliorating oxidative stress and preventing tumour growth in the human gastrointestinal tract. Future research efforts should therefore be directed towards the development of effective rice bran dietary interventions, and the assessment of their effectiveness in reducing the presence of biomarkers indicative of both oxidative stress and tumour development among gastrointestinal cancer survivors. Such studies may not only provide further evidence for the effect of rice bran intake in gastrointestinal cancer prevention through the modulation of oxidative stress, but also establish the potential for using rice bran dietary interventions in inhibiting the recurrence of gastrointestinal cancer among survivors of the disease.

## Figures and Tables

**Table 1:** A summary of the studies showing the anti-oxidative effects of rice bran.

Supplementation/Treatment	Model	Effects of Supplementation/Treatment	Reference
Rice bran	Rat	Increase in GST activity	Boetang et al. 2009 [36]
Enzymatic extract of rice bran	Rat	Increase in activity of liver SOD, GPx and catalase Decrease in MDA and protein carbonyl levels in liver	Wang et al. 2014 [37]
Enzymatic extract of rice bran	Rat	Decrease in NADPH oxidase expression Decrease in superoxide production	Justo et al. 2013 [38]
Ethanolic extract of rice bran	HL-60 Leukaemia cell line	Decrease in superoxide production Decrease in MDA levels	Hansakul et al. 2011 [39]
Methanolic extract of rice bran	C6 glioma cell line	Ability of the extract to scavenge ROS and nitric oxide, and this effect was dose-dependent	Rao et al. 2010 [40]
Methanolic extract of rice bran	HepG2 Liver cancer cell line	Decrease in ROS production Decrease in MDA levels Prevention of glutathione depletion	Lee et al. 2014 [41]
Methanolic extract of rice bran	H9c2 (2-1) Rat cardiomyocytes	Increase expression level and activity of catalase	Tan et al. 2016 [43]
Fermented extract of rice bran	3T3-L1 adipocytes	Inhibition of ROS generation	Kim and Han, 2011 [42]
Rice bran oil	Rat	Reduction of MDA levels Slightly increase the activity of SOD, catalase and GPx	Sengupta et al. 2014 [44]
Rice bran oil	Rat	Increase in GST activity	Panala et al. 2009 [46]
Rice bran oil	Rat	Decrease in 8-oxodG level in mitochondria in the liver, pancreas and kidney	Hsieh et al. 2005 [47]
Rice bran oil (tocotrienol fraction)	Rat	Increase in GST activity Reduction of lipid peroxidation and low-density lipoprotein oxidation	Iqbal et al. 2004 [48]
Riceberry bran oil	Rat	Decrease in MDA levels Increase in the activity of SOD and GPx	Posuwan et al. 2012 [45]
MGN3/Biobran	Mouse	Increase in glutathione levels Increase in the expression and activity of SOD, catalase, GPx and GST	Noaman et al. 2008 [49]

Abbreviations: GPx, glutathione peroxidase; GST, glutathione-S-transferase; MDA, malondialdehyde; NADPH, reduced nicotinamide adenine dinucleotide phosphate; ROS, reactive oxygen species; SOD, superoxide dismutase.

**Table 2:** A summary of the evidence for the anti-oxidative effects of rice bran phytochemicals in cancer prevention.

Phytochemical	Model	Anti-Oxidative Effect of Phytochemical	Evidence for Its Ability of Cancer Prevention	Reference
Phytic acid	Rat model of liver cancer	Increase in GST activity Reduction in lipid peroxidation	Decrease in the level of placental GST-positive foci, a marker of hepato-carcinogenesis	Lee et al. 2005 [56]
Phytic acid	Rat model of CRC	Increase in GST activity	Decrease in the number of colon tumours	Khatiwada et al. 2011 [57]
Ferulic acid	Rat model of CRC	Increase in GST activity	Decrease in the number of aberrant crypt foci	Kawabata et al. 2000 [58]
p-Methoxycinnamic acid	Rat model of CRC	Reverse the effect of chemically-induced CRC in rats, by reducing level of lipid peroxidation and protein oxidation in liver Increase in activity of SOD, catalase and GPx Increase in glutathione, vitamin E and vitamin C levels	Decrease in the number of aberrant crypt foci and colon tumours	Sivagami et al. 2012 [60]

**Abbreviations:** CRC, colorectal cancer; GPx, glutathione peroxidase; GST, glutathione-S-transferase; SOD, superoxide dismutase.
